# Assistive technologies for people with dementia: ethical considerations

**DOI:** 10.2471/BLT.16.187484

**Published:** 2017-05-12

**Authors:** Belinda Bennett, Fiona McDonald, Elizabeth Beattie, Terry Carney, Ian Freckelton, Ben White, Lindy Willmott

**Affiliations:** a Australian Centre for Health Law Research, Queensland University of Technology, 2 George Street, Brisbane, Queensland, 4000, Australia.; b Dementia Collaborative Research Centre, Queensland University of Technology, Brisbane, Australia.; c Law School, University of Sydney, Sydney, Australia.; d Law School, University of Melbourne, Melbourne, Australia.

## Abstract

The sustainable development goals (SDGs) adopted by the United Nations in 2015 include a new target for global health: SDG 3 aims to “ensure healthy lives and promote well-being for all at all ages.” Dementia care of good quality is particularly important given the projected increase in the number of people living with the condition. A range of assistive technologies have been proposed to support dementia care. However, the World Health Organization estimated in 2017 that only one in 10 of the 1 billion or more people globally who could benefit from these technologies in some way actually has access to them. For people living with dementia, there has been little analysis of whether assistive technologies will support their human rights in ways that are consistent with the United Nations Convention on the Rights of Persons with Disabilities. The aim of this paper is to examine the relevant provisions of the convention and consider their implications for the use of assistive technologies in dementia care. Assistive technologies can clearly play an important role in supporting social engagement, decision-making and advance planning by people living with dementia. However, concerns exist that some of these technologies also have the potential to restrict freedom of movement and intrude into privacy. In conclusion, an analysis of the implications of assistive technologies for human rights laws is needed to ensure that technologies are used in ways that support human rights and help meet the health-related SDG 3.

## Résumé

Les objectifs de développement durable (ODD) adoptés par les Nations Unies en 2015 intègrent un nouvel objectif de santé mondiale: l'ODD3, qui vise à « permettre à tous de vivre en bonne santé et promouvoir le bien-être de tous, à tout âge ». Une prise en charge de bonne qualité de la démence est particulièrement importante, compte tenu des projections d'augmentation du nombre de personnes atteintes. Plusieurs technologies d'assistance sont proposées pour contribuer à une bonne prise en charge de la démence. Néanmoins, en 2017, l'Organisation mondiale de la Santé a estimé qu'à l'échelle mondiale, parmi le milliard de personnes -voire plus- susceptibles de bénéficier de ces technologies, seule une personne sur 10 peut effectivement y avoir accès. Peu d'analyses ont été réalisées pour déterminer si l'utilisation des technologies d'assistance pour les personnes atteintes de démence peut contribuer à l'application des droits de l'homme au sens de la Convention des Nations Unies relative aux droits des personnes handicapées. L'objectif de cet article consiste à examiner les dispositions pertinentes de cette convention et à considérer leurs implications pour l'utilisation des technologies d'assistance dans la prise en charge de la démence. Il est clair que les technologies d'assistance peuvent jouer un rôle important pour aider à l'implication sociale, à la prise de décision et à la planification anticipée des soins chez les personnes atteintes de démence. Mais des doutes existent aussi sur le potentiel de certaines de ces technologies à restreindre la liberté de mouvement et à enfreindre la vie privée. En conclusion, il est nécessaire d'analyser les implications de l'usage des technologies d'assistance à la lumière des législations sur les droits de l'homme, afin de s'assurer que ces technologies soient employées de manière à contribuer à l'application des droits de l'homme et à atteindre l'ODD3 lié à la santé.

## Resumen

Los objetivos de desarrollo sostenible (ODS) adoptados por las Naciones Unidas en 2015 incorporan un nuevo objetivo para la salud mundial: el ODS 3 tiene como objetivo “garantizar vidas sanas y promocionar el bienestar para todos y para todas las edades”. Una atención a la demencia de buena calidad es especialmente importante dado el aumento previsto en el número de personas con dicha enfermedad. Se ha propuesto una gama de tecnologías de asistencia para apoyar la atención a la demencia. Sin embargo, la Organización Mundial de la Salud estimó en 2017 que solo una de cada 10 personas de los mil millones de habitantes o más que hay en el mundo que podrían beneficiarse de tales tecnologías en cierto modo realmente pueden acceder a ellas. Para las personas con demencia, se han realizado pocos análisis de si las tecnologías de asistencia apoyarán sus derechos humanos de forma coherente con la Convención de las Naciones Unidas sobre los Derechos de las Personas con Discapacidad. El objetivo de este artículo es examinar las disposiciones más importantes de la convención y considerar las consecuencias del uso de tecnologías de asistencia para la atención a la demencia. Sin duda, estas tecnologías pueden tener un papel importante a la hora de apoyar el compromiso social, la toma de decisiones y la planificación adelantada por parte de las personas que sufren demencia. Sin embargo, existen temores de que algunas de estas tecnologías puedan limitar la libertad de movimiento e invadir la privacidad. En conclusión, es necesario realizar un análisis de las consecuencias de las tecnologías de asistencia sobre las leyes de derechos humanos para garantizar que se utilicen las tecnologías de forma que respalden los derechos humanos y ayuden a lograr el ODS 3 relacionado con la salud.

## ملخص

تشتمل أهداف التنمية المستدامة (SDGs) التي اعتمدتها الأمم المتحدة في عام 2015 على هدف جديد للصحة العالمية: إذ يرنو الهدف الثالث من أهداف التنمية المستدامة إلى "ضمان تمتّع الجميع بأنماط عيش صحية وبالرفاهية في جميع الأعمار". وتعتبر الرعاية عالية الجودة للخَرَف أمرًا بالغ الأهمية نظرًا للزيادة المتوقعة في عدد المصابين بهذه الحالة. وقد تم اقتراح مجموعة من التقنيات المساعدة لدعم رعاية الخَرَف. على الرغم من ذلك فقد أشارت تقديرات منظمة الصحة العالمية في عام 2017 إلى أن نسبة القادرين على الاستفادة من تلك التقنيات قد اقتصرت على واحد فقط من أصل كل عشرة من بين المليار نسمة أو أكثر على مستوى العالم ممن توفرت لهم سبل الاستفادة من هذه التقنيات بطريقة أو بأخرى. وبالنسبة للمصابين بالخَرَف فقد كانت هناك تحليلات محدودة لما إذا كانت التقنيات المساعدة سوف تدعم حقوقهم الإنسانية بطرق تتوافق مع اتفاقية الأمم المتحدة بشأن حقوق ذوي الإعاقات. ويهدف هذا التقرير إلى دراسة نصوص الاتفاقية ذات الصلة وأخذ آثارها بعين الاعتبار لتوظيف التقنيات المساعدة في رعاية الخَرَف. ويمكن أن تلعب التقنيات المساعدة وبشكل واضح دورًا هامًا في دعم المشاركة الاجتماعية، واتخاذ القرار، والتخطيط المسبق للمصابين بالخَرَف. وعلى الرغم من ذلك فهناك مخاوف من أن بعض هذه التقنيات تواجه أيضًا احتمال تقيد حرية الحركة والتدخل في الخصوصية. في الختام فإننا بحاجة إلى تحليل آثار التقنيات المساعدة على قوانين حقوق الإنسان وذلك لضمان توظيف هذه التقنيات بطرق تدعم حقوق الإنسان وتتماشى مع الهدف الثالث للتنمية المستدامة المعني بالرعاية الصحية.

## 摘要

联合国 2015 年可持续发展目标 (SDG) 中包括一项全球健康新目标： SDG 3 目标致力于“保证人们的健康生活和促进所有年龄段人们的健康。” 预计增加的患病人数使得优质的痴呆症护理尤为重要。 已提出一系列辅助技术用以支持痴呆症护理。 然而，世界卫生组织预计，在 2017 年，全球可通过某种方式从辅助技术中获益的 10 亿或更多的人中，只有十分之一的人能真正获取这些技术。 对于痴呆症患者而言，几乎没有任何关于辅助技术是否在符合《联合国残疾人权利公约》的前提下支持其人权的分析。 本文的目的是审视相关公约规定，并考虑其对痴呆症护理中使用辅助技术的影响。 辅助技术在支持痴呆症患者的社会参与、决策和提前规划方面可以发挥显著的重要作用。 然而，对于某些辅助技术有可能限制行动自由和侵犯隐私，仍存在担忧。 总之，需要对辅助技术对人权法律的影响进行分析，以确保在支持人权的前提下使用技术并帮助实现健康相关 SDG 3 目标。

## Резюме

Цели в области устойчивого развития (ЦУР), принятые Организацией Объединенных Наций в 2015 году, включают новую цель международного здравоохранения: ЦУР 3 направлена на «обеспечение здоровой жизни и повышение благосостояния каждого человека в любом возрасте». Наиболее важным является высокое качество медицинского обслуживания при деменции с учетом прогнозируемого увеличения числа людей, живущих с этим заболеванием. Был предложен ряд вспомогательных технологий в поддержку медицинского обслуживания при деменции. Однако по оценке Всемирной организации здравоохранения, сделанной в 2017 году, среди 1 миллиарда человек в мире, которые могли бы воспользоваться этими технологиями, только один из 10 человек фактически имеет к ним доступ. Для людей, живущих с деменцией, был проведен небольшой анализ того, будут ли вспомогательные технологии поддерживать их права человека способами, которые согласуются с Конвенцией Организации Объединенных Наций о правах инвалидов. Цель настоящего документа — изучить соответствующие положения конвенции и рассмотреть последствия использования вспомогательных технологий в медицинском обслуживании при деменции. Вспомогательные технологии, несомненно, могут играть важную роль в поддержке социальной интеграции людей, живущих с деменцией, а также обеспечить им поддержку при принятии решений и перспективном планировании. Однако существуют опасения, что некоторые из этих технологий могут также ограничивать свободу передвижения и вмешиваться в частную жизнь. В заключение нужно отметить, что необходимо проанализировать последствия внедрения вспомогательных технологий относительно законодательства в области прав человека для обеспечения того, чтобы используемые технологии поддерживали права человека и способствовали выполнению ЦУР 3, относящейся к здравоохранению.

## Introduction

The sustainable development goals (SDGs) adopted by the United Nations in 2015 propose a new target for global health: the aim of SDG 3 is to “ensure healthy lives and promote well-being for all at all ages”.^1^ This paper addresses the role of technology in helping to achieve this goal. It does so by focusing on how assistive technologies can help improve the well-being of people living with dementia and by placing a particular emphasis on the human rights implications of these technologies.

There are several reasons for this approach. First, although assistive technologies can play a vital role in supporting the well-being of people with disabilities, there is a huge unmet need for access to these technologies. According to World Health Organization (WHO) estimates, more than a billion people globally could benefit from assistive technologies, yet only one in 10 has access to them.^2^ Research into how assistive technologies can improve well-being will help raise awareness of this need. Second, although assistive technologies may support people with a wide range of disabilities, a focus on care for elderly people is relevant given that the population is ageing in many countries. As WHO has noted, due to ageing populations and increases in noncommunicable diseases, more than 2 billion people will need at least one assistive technology by 2050.^2^ Third, dementia is an important public health concern in ageing populations. In 2012, WHO concluded that “dementia poses one of the greatest societal challenges for the 21st century”.^3^ In many countries, an ageing population will be associated with a disproportionately large rise in the number of people living with dementia – the number may increase from 46.8 million people worldwide in 2015 to an estimated 131.5 million by 2050,^4^ with substantial implications for health-care costs. The total worldwide cost of dementia in 2018 could be 1 trillion United States dollars.^4^ Fourth, without research into assistive technologies and greater global awareness of the unmet need for these technologies, particularly in low- and middle-income countries,^5^^,^^6^ the divide might grow between countries in access to assistive technologies based on their income.^2^ Finally, an emphasis on human rights provides a reminder that technologies should be introduced in ways that are supportive of and respect the rights of vulnerable members of the community.^7^

## Assistive technologies

Assistive technologies range from basic aids, such as walking canes, through to high-tech devices such as robots.^5^^,^^6^ Some new assistive technologies are potentially valuable for improving the health and care of people living with dementia: memory and communication aids, safety devices,^8^ global positioning system (GPS) tracking,^9^^–^^11^ companion robots^12^^–^^14^ and technology for so-called smart homes.^15^^,^^16^ Indeed, it has been argued that these technologies may have a triple-win effect because they may: (i) delay entry into institutional care; (ii) reduce the burden on caregivers; and (iii) improve the quality of life of dementia patients.^17^ The use of assistive technologies is often limited to basic aids but older people’s growing familiarity with the Internet and increased global use of digital technologies, including mobile phones, may help drive demand for more sophisticated aids.^5^

Although the ethical implications of assistive technologies have previously been examined,^18^^–^^21^ very little consideration has been given to legal issues,^22^ to how these technologies could or should be regulated,^15^ or to the measures that may need to be taken to ensure their use is consistent with human rights obligations and with social citizenship rights of participation in civil society. Moreover, little attention has been paid to incorporating decisions about assistive technologies into the advance care planning processes, even though, as Eltis points out, “decisions respecting assistive technologies can and should be made at the time of diagnosis”.^22^

Traditionally, concerning behaviour by people with a cognitive impairment, including dementia, has been dealt with using restrictive practices, such as physical restraint or medication.^7^^,^^23^^–^^25^ The use of assistive technologies in dementia care adds a new technological dimension: in addition to supporting people living with dementia and enhancing their living arrangements, assistive technologies may also provide new mechanisms for surveillance, for limiting privacy and for restricting movement.

The use of robots in dementia care may help reduce loneliness in some people living with the condition, may provide opportunities for social interaction and may ease the burden of care, which is traditionally borne by women.^26^ However, it has also been argued that social robots may devalue caring and reduce human contact with the elderly, thus limiting rather than enhancing social interaction.^26^ For some, the use of robotics in dementia care may be seen as part of a broader social trend to outsource care, a trend that undermines the concept that a person-to-person relationship is the foundation of care and that, it is argued, risks transforming the nature of the care encounter.^27^ Complex ethical questions also arise. For example, when a human interacts with a machine that has human characteristics and treats that machine as if it were human, does this pose a problem, particularly for vulnerable people? And, is there a level of deception involved that makes the use of such machines unethical?^28^^,^^29^ The ongoing debate on whether assistive technologies will enhance or undermine the dignity of those living with dementia^18^ makes it essential that the human rights implications of these technologies are examined.

## Human rights

In a conference briefing document published in 2015, WHO recognized the importance of a human rights-based perspective on people living with dementia and recommended adopting the PANEL+ approach, which addresses participation, accountability, non-discrimination, empowerment and legality.^7^ Patients’ organizations can help ensure human rights and the first international organization whose members comprise only people with dementia – Dementia Alliance International – was established in 2014.^30^ In addition, the United Nations Convention on the Rights of Persons with Disabilities^31^ is critically important for developing a robust, rights-focused regulatory framework for the use of assistive technologies in dementia care. Governments that are signatories to the convention are required to adopt a human rights framework and it is expected that the convention will have a profound impact on countries’ mental health and disability laws.^32^ It has been suggested that ratification of the convention would be a good starting point for jurisdictions that do not have domestic legislation supporting the use of assistive technologies for people with disabilities.^5^^,^^6^

According to McSherry, the convention “has the potential to change the landscape for human rights for those with disabilities”.^33^ Importantly, the convention is not simply aspirational but “is a formal convention, not mere guidance, and therefore has the force of international law”.^32^ The requirement in the convention for States Parties to provide reports on their domestic implementation of the convention (Article 35) and to make these reports publically available (Article 36) means that actions taken to implement the convention will be subject to public, international scrutiny.^32^^,^^34^ Although the convention has a transformative potential for the care of people with disabilities in general, several of its provisions have particular relevance to dementia care.

### Article 4

Under Article 4 of the convention, States Parties have an obligation to undertake research and to develop new technologies, including assistive technologies, “suitable for persons with disabilities, giving priority to technologies at an affordable cost” – Article 4(1)(g) – and to provide information about such technologies – Article 4(1)(h). These provisions recognize that assistive technologies can improve the lives of people with disabilities, including those with dementia. They are also consistent with the central role governments must play in addressing the unmet need for assistive technologies. Importantly, the requirement under Article 5(3) for States Parties to ensure that reasonable accommodation is provided for people with disabilities imposes an obligation on States Parties to provide positive support. The provision, availability and use of assistive technologies to support people living with dementia may, then, fall within the reasonable accommodation requirement.

### Article 9

Assistive technologies may also help people with disabilities, including dementia, to continue to live independently. Obligations under convention Article 9 address accessibility – that is, provisions that “enable persons with disabilities to live independently and participate fully in all aspects of life”.

### Article 12

Article 12 of the convention requires that people with disabilities have “equal recognition before the law”. Much of the analysis of the convention has focused on one particular implication of Article 12:^35^^,^^36^ the move towards supported decision-making and the need to respect the rights, will and preferences of people living with cognitive impairment and away from the traditional approach of substitute decision-making, where decisions are made for others. States Parties may therefore need to amend their domestic legislation to ensure that the legal rights of people living with dementia are recognized. As WHO has noted, legislation should recognize “free and informed consent to treatment, supported decision-making, and procedures for implementing advance directives”.^7^

### Article 18

Under Article 18 of the convention, States Parties are required to recognize, among other things, the rights of people with disabilities to liberty of movement and to choose their residence freely. These requirements have implications for the development of national laws and policies to support people with disabilities, including people living with dementia, and to recognize the need for dementia-friendly environments that support healthy and active ageing.^7^ Entitlements that affect where a person can go or live are among the most sensitive for people living with dementia. Technologies such as GPS tracking that can be used in and beyond the home may enable people with dementia to enjoy greater freedom of movement and to stay in their home longer than would otherwise be possible.

### Article 19

Article 19 of the convention supports the right to live independently and to be included in and participate in the community. However, the degree to which assistive technologies will facilitate these rights is unclear at present. For example, although assistive technologies may support family members and other informal carers and may enable people with disabilities to continue being cared for at home rather than in an institution, it is unclear whether these technologies will actually broaden social interactions. Further research is required in this area. For instance, it has been argued that Article 19 should be interpreted as implying that barriers and obstacles to therapeutic access to outdoor spaces should be eliminated. The removal of such barriers would reduce the adverse impact that confinement within closed spaces, often indoors, may have upon a progressive condition such as dementia.^37^

### Article 22

Electronic and other forms of monitoring and surveillance can be used to ensure the safety and well-being of people living with dementia. However, their use may intrude into people’s privacy to an extent that, on balance, may not be justifiable and may even have negative therapeutic consequences because of their impact on a person’s sense of independence and autonomy. National laws should protect the right to privacy of people living with dementia – this right is recognized in convention Article 22, which precludes people with disabilities from being subjected to various forms of unacceptable interference with their privacy, correspondence or communications. Moreover, there is an express stipulation that this right should apply “regardless of place of residence or living arrangements”.

### Article 25

Article 25 of the convention recognizes the health-related rights of people with disabilities and the “right to enjoyment of the highest attainable standard of health without discrimination on the basis of disability”. As we have argued, various kinds of assistive technologies have the potential to improve the health of people living with dementia and access to these technologies, where beneficial, is essential for achieving the health-related SDG 3.

Without an evaluation of assistive technologies’ implications for human rights, they may fail to live up to their promise to improve the lives of people with dementia and their carers. They may even have a counterproductive effect, by eroding rights and freedoms that are highly valued by people living with dementia. A final consideration, over and above the principles articulated in the convention, is the need for distributive justice and fair access to assisted technologies by people with dementia.^17^ Given the unmet need for assistive technologies, particularly in low- and middle-income countries, the promotion of innovation and the development of low-cost assistive technologies should be prioritized.^5^^,^^6^^,^^38^

## Technological development

The development of assistive technologies involves research to evaluate the needs and wishes of people living with dementia and their carers to assess the acceptability and utility of these technologies.^39^ The participation of people with dementia is required to obtain meaningful results,^40^ which links to the theme of participation in WHO’s PANEL+ approach to dementia.^7^

Dementia strikes at the heart of the traditional principles guiding participation in research, namely the requirement to obtain an individual’s consent. Such consent depends on the person having decisional capacity.^41^ Until relatively recently, people perceived to be vulnerable, especially those with cognitive impairments such as dementia, who may be incompetent to take decisions, have often been excluded from participating in research to protect them from exploitation.^40^^,^^42^ In the face of concerns that exclusion from research was protecting some groups to death, however, vulnerable groups are no longer routinely excluded from the benefits that can accrue from participating in research.^40^^,^^42^ The evolution from substituted decision-making to supported decision-making under the convention raises questions about how consent for people living with dementia to participate in dementia-focused research should be negotiated in the future. Although the ability of people living with dementia to make decisions about participation should not be underestimated,^41^ it is still not clear exactly what type of consent to research for people living with dementia will be negotiated under a convention-focused framework for decision-making.

The need for, or adoption of, assistive technologies may be influenced by other technological developments in society. For example, many people living at home with early-stage dementia provide their internet banking access details to a family member or friend, who then manages their finances with them on an informal basis. Technological advances, such as the use of iris identification instead of passwords, may undermine the utility of such arrangements (which are not free of the risk of misuse). This may result in more restrictive options: for example, the person living with dementia: (i) may have to officially appoint payment nominees or representative payees for pensions;^43^ (ii) may be pressured to execute enduring or durable powers of financial attorney; or (iii) may have to resort to formal financial guardianship. This would be problematic under the convention, which rejects proxy decision-making.^44^ On the other hand, the development of smart card technologies may offer better guarantees of personal privacy than are available under protective regimes that require the registration of durable powers as a condition of their recognition – a measure which may deter the adoption of such private planning options.^45^

## Advance care planning

Technology can also be used to increase awareness of the need for advance care planning (for example, via social media) and to improve access to documentation on planning. In addition, technology has changed the way this documentation is stored and retrieved – the effect has been to enhance the reliability, accessibility and (perceived) authority of advance care plans, for example, through e-health initiatives.^46^ The process of advance care planning could also be improved by technology. Although the literature on decision-making aids in health care^47^ and in advance care planning is extensive,^48^ there has been very little research on how assistive technologies could be used in such planning.

A key question is whether assistive technologies could extend the period during which a person living with dementia would have the capacity to make their own decisions about health care. Technologies that support memory, provide a concrete decision-making process that organizes complex decisions into a series of more manageable options, or provide medical information to help frame choices are examples of technologies that can assist the supported decision-making required by the convention, according to at least one understanding of supported decision-making. These technologies – and others that facilitate communication – could also be relied upon to help discern a person’s will and preferences in relation to health care for a longer time than current law recognizes him or her as having capacity. Under Article 12, the convention requires a person’s will and preferences to be respected despite cognitive impairment. However, this requirement may give rise to tensions with other rights, including the right to health recognized in Article 25. One issue yet to be properly resolved is whether or not this conception of supported decision-making should be recognized when the decision being made is one that will lead to withholding or withdrawing life-sustaining treatment, thus leading to the person’s death.

## Conclusion

Assistive technologies can play an important role in supporting both the independence and care of people living with dementia and in helping them live healthy lives, as encapsulated in SDG 3. However, to meet this goal, it is essential that assistive technologies are affordable and accessible for people living with dementia and their carers and that staff charged with helping with the use of these technologies are trained and capable. The achievement of universal health coverage (target 3.8 of SDG 3) will help to ensure that the necessary technologies are accessible and affordable. For low- and middle-income countries, WHO’s *Priority assistive products list*^49^ will help countries meet their obligations under the SDGs and the Convention on the Rights of Persons with Disabilities. Furthermore, although assistive technologies may support independence, some technologies have the potential to intrude upon the rights, privacy and freedom of people living with dementia. Consequently, careful consideration should be given to their implications for human rights, in particular whether they support the principles articulated in the convention, which provides an established and valuable human rights framework for appraising new technologies. An approach to evaluating innovative technologies that places human rights at its core will help ensure that these technologies will improve the lives of people living with dementia.
